# Effectiveness of the mixture of nopal and cassava starch as clarifying substances in water purification: A case study in Colombia

**DOI:** 10.1016/j.heliyon.2020.e04296

**Published:** 2020-06-26

**Authors:** José Lugo-Arias, Elkyn Lugo-Arias, David Ovallos-Gazabon, Juan Arango, Mario de la Puente, Jesús Silva

**Affiliations:** a Universidad de Cundinamarca, Colombia; b Corporación Universitaria Minuto de Dios Colombia, Colombia; c Universidad Simón Bolívar, Colombia; d Department of Political Science, Universidad del Norte, Colombia; e Universidad Peruana de Ciencias Aplicadas, Perú

**Keywords:** Nopal mucilage, Cassava starch, Natural coagulant, Flocculating aid, Magdalena, Drinking water treatment, Chemical engineering, Water treatment, Environmental science, Environmental engineering, Environmental health

## Abstract

Aluminum sulfate is one of the most used chemical coagulants in the world, but research has shown that high concentrations of aluminum in the body are associated with neuropathological conditions. Because of this, different alternatives have been evaluated such as natural coagulants, which are considered safe for human health and contain fewer contaminants than chemicals due to their biodegradation properties. The main objective of this study was to evaluate the efficiency of mixing nopal mucilage and cassava starch for turbidity removal in water purification. In this paper, test jars and the treatment equipment (TA-scale FQ-005/PE manufactured by Generatoris SA de CV of Mexico) was applied in order to measure turbidity and pH parameters before and after the process of coagulation–flocculation, which was applied to water from the Magdalena River in Colombia. Samples from two sampling periods were assessed. One was evaluated during the rainy season and the other was evaluated without precipitation (drought) with initial turbidities of 316 NTU and 80 NTU, respectively. It was found that aluminum sulfate as a coagulant reference obtained better turbidity removal results (up to 99%) as compared to nopal (up to 60.4%), and nopal–starch combination of cassava (up to 67%), indicating that this mixture increases the effectiveness of natural coagulants used individually. Our results indicate that this should be considered as an alternative in the water purification process.

## 1 Introduction

High residual concentrations of aluminum in drinking water can be traced back to the utilization of aluminum sulfate, one of the most used chemical coagulants in the world, in the water treatment process; high concentrations of aluminum are associated with brain injury in humans (Manivannan et al., 2015; Chen et al., 2016). To avoid these adverse effects on human health, it is necessary to comply with regulations that define the maximum permissible limit of contaminants, such as residual aluminum, in drinking water; for example, the Environmental Protection Agency sets a maximum concentration of 0.2 mg/L for aluminum in drinking water (Freitas et al., 2016).

Additionally, it is important to take the chemical speciation of aluminum in the treatment of drinking water into account since the form of aluminum controls its solubility, bioavailability, and toxicity; especially given that aluminum salts (e.g., aluminum sulfate) can increase the percentage of dissolved forms of low molecular weight aluminum, which are chemically reactive, more easily absorbed by the human body, and could cause neuropathological effects (Krupińska, 2020).

Due to these concerns, researchers are assessing potential water treatment alternatives, such as natural coagulants, which are considered safe for human health and less contaminating than chemicals as a result of their biodegradation properties (Freitas et al., 2015).

Numerous studies have evaluated the use of natural coagulants as a strategy to replace chemical coagulants that are commonly used in water treatment. Natural compounds that have been found to be highly effective include *Moringa oleifera* (Camacho et al., 2017; Lugo-Arias et al., 2020), *Plantago ovata* (Dhivya et al., 2017), pine cone (Hussain et al., 2019), and mucilage nopal (Torres et al., 2012).

The prickly pear (*Opuntia ficus-indica*) is a member of the cactus family, which is native to the Americas and has many industrial uses. Besides its application as a coagulant, it is highlighted with an average efficiency of 90% with regard to water turbidity removal (Flores et al., 2006). Olivero et al. (2013) conducted a study in the city of Arjona (Bolívar, Columbia) regarding the efficiency of nopal against aluminum sulfate and found that nopal was 93.25% effective in removing turbidity compared to aluminum sulfate, which had the highest efficacy concerning turbidity removal (99.80%).

Given the above study, aluminum sulfate is more efficient as a coagulant than mucilage nopal. However, flocculation assistants also play a key role in the water treatment processes, therefore, it is also important to identify natural substances that can increase the efficiency of the coagulation–flocculation process.

Flocculation assistants, also known as flocculants and flocculation aids, are colloids that are added during the solid–liquid separation processes to help form heavier flocs. Some flocculation aids, such as polyacrylamide, are synthetic derivatives (Ramírez Arcila and Jaramillo Peralta, 2015). However, there are also natural flocculation assistants such as cocklebur, balsa, and guásimo, which have achieved efficiencies of 93.6%, 90.4%, and 89.7%, respectively in the clarification processes of cane juice (Ortiz et al., 2011). These three flocculants were applied in concentrations that varied between 200 and 300 mg/L, which were higher concentrations compared to polyacrylamide that has a reported range between 0.05 and 0.5 mg/L. Chemical or synthetic flocculants are generally applied in lower doses than biologically based flocculants such as those based on starch. For example, cassava starch is typically applied in concentrations between 5 to 20 mg/L, as confirmed in Lapointe and Barbeau (2017), Lapointe and Barbeau (2015), and Lapointe and Barbeau (2019). Perhaps previously mentioned high doses limit the application of these natural substances in water treatment for human consumption, thus, it is important to evaluate and establish mechanisms to take advantage of the active compounds in natural flocculants that are most effective for coagulation–flocculation processes.

Meanwhile, cassava starch achieved removals of 75% of haze and 78% of color in domestic wastewater when used in combination with aluminum sulfate (alum) (Ortiz et al., 2018); whereas a study by Solís et al. (2012) found removals of 94% of haze in river water using the same coagulant mixture. Likewise, Contreras et al. (2015) achieved removals of 98% of turbidity and 100% of color when using nopal mixed with alum. These combinations are interesting because natural substances mixed with a chemical reduce the concentration of chemical components that may have deterimental effects on human health, as mentioned with respect to aluminum sulfate. Studies focusing on combinations of chemical coagulants and natural flocculants are highly relevant to explore in the scientific field. It would be appropriate to analyze coagulants and natural flocculants since they are considered safe for human health; and thus, the use of synthetic and potentially toxic chemicals in water clarification processes would be reduced or ultimately replaced.

However, a study evaluating the combination of nopal as a coagulant and cassava starch as a flocculating aid has not been developed, as mentioned in literature reviews made by Jayalakshmi et al. (2017) and Saleem and Bachmann (2019), even though it is a sustainable water treatment alternative. Here, our study assesses the efficacy of the mucilage mixture of nopal and cassava starch in removing turbitity during water treatment processes.

## 2 Materials and methods

### 2.1 Sample preparation

In this project, two samples were taken from the Magdalena River in the city of Girardot, Colombia, 1.3 km downstream at the mouth of the Bogota River to the Magdalena River. One sampling session was done on a rainy day (April 23, 2019) and the other on a day without precipitation (May 7, 2019). Specifically, the sampling point was at the pier of Girardot (latitude 4° 17′ 38.5″N and longitude 74° 48′ 37.7 l" W). During each sampling period, 100 L of problem water were taken and analyzed the same day in the water laboratory at the University of Cundinamarca Sectional Girardot.

### 2.2 Analytical methods for water quality

The parameters evaluated were: 1) turbidity using a portable turbidity meter (Hanna Instruments, model HI 93703) and 2) pH with a pH meter (Hanna Instruments, model HI 98127). Measurement of these parameters was performed with consideration to the guidelines of Standard Methods for the Examination of Water and Wastewater: Turbidity (2130, nephelometric) and pH (H + B 4500, electrometric). The quality of the data analyzed in the laboratory was guaranteed by procedural blank measurements, making triplicates for each parameter of water quality evaluated, and recording the average values of pH and turbidity.

### 2.3 Methodological design

The work was conducted in two stages. The first stage concerned the methodology for the preparation of the coagulant and flocculant to be evaluated; and the second stage was based on laboratory tests to determine the efficiency of natural substances evaluated in the removal of turbidity from the surface water source being studied.

#### 2.3.1 Preparation of coagulant and flocculant

The extraction of mucilage from nopal (coagulant) was performed taking into account the methodology of Contreras et al. (2015) and Alimi et al. (2010). The procedure was as follows: nopal stalks were selected, washed, and cut into cubes for easy grinding in a household blender, and distilled water was added in proportion 1:2 (v/v). The obtained mixture underwent a heating treatment in an oven at 50 °C for one hour. After this, suspension was centrifuged at 3500 rpm for 10 min. The pellet was discarded and ethanol was added to the supernatant (1:4 v/v) and was allowed to stand for 24 h under refrigeration at 5 °C to precipitate the mucilage. The precipitated mucilage dried without prior filtration, evaporating at 70 °C until it reached a maximum moisture content of 10%. Finally, the product obtained was pulverized using a mortar.

The cassava starch (flocculant), which presents as a fine white powder, was bought in a store (Salsamentaria) located in the market square of the municipality of Girardot, Colombia.

#### 2.3.2 Tests effectiveness of natural substances studied

We determined the effectiveness of the coagulants evaluated and their combination with the flocculating aid using test jars and the equipment of a small-scale physical–chemical treatment from Mexico (TA- FQ-005/PE manufactured by Generatoris SA de CV). The evaluation of the natural compounds being studied was conducted in two study seasons (wet and dry).

As a first step, three 1% mother solutions were prepared with distilled water: the nopal, cassava starch, and aluminum sulfate ($Al_{2}{\left(SO_{4}\right)}_{3}$) commercial type A as a reference. After obtaining these solutions, test jars were applied in which rapid mixing was conducted with an agitation rate of 100 rpm per minute, followed by slow mixing at 50 rpm for 20 min, and finally the samples were allowed to stand for 20 min with the jar equipment turned off. Upon completion of the jar test, water samples were taken in each of the beakers, stirred, and measured for turbidity and pH values. We obtained data that compared the initial values of these two parameters using the following relationship, which sets the percentage removal of each coagulant and flocculant used, respectively:

(1) $$Efficiency\left(\%\right)=\frac{initial\;concentration-final\;concentration}{initial\;concentration}*100\;$$

The applied doses of aluminum sulfate to the water being studied were 25 mg/L, 35 mg/L, 50 mg/L, 80 mg/L, 100 mg/L, and 160 mg/L. As for the evaluation of nopal, concentrations were used within the range previously evaluated by Contreras et al. (2015) and Miller et al. (2008), which are: 15 mg/L, 25 mg/L, 35 mg/L, 45 mg/L, 55 mg/L, 70 mg/L, and two additional concentrations of 100 mg/L and 150 mg/L, respectively.

Subsequently, the determined optimal dose of the mucilage nopal tested in combination with cassava starch as a flocculation assistant was used to assess whether cassava starch increases the effectiveness of nopal as a coagulant. The proportions of the combinations were based on research by Solís et al. (2012), but unlike this project, his study concerned the use of aluminum sulfate in combination with cassava starch. The percentages of respective combinations of nopal–cassava starch were: a) 93% and 7%; b) 85% and 15%; c) 75% and 25%; d) 72% and 27%; e) 67% and 33%; and f) 60% and 40%.

Finally, the combinations of coagulant and natural flocculant were evaluated using the TA- CF-005/PE equipment. The test with this kit was developed following a practical manual, which consisted of flow variation of pumped raw water, a solution of coagulant and flocculant for 60 min, and measuring turbidity values at the beginning and end of the process in order to simulate the actual conditions of a water treatment plant. Coagulant flow rates varied from 0.3 to 3 L/h, the flow rate of flocculant ranged between 0.1 and 2 L/h, and the problem of water was between 5 and 12 L/h. In all variations, a 1% stock solution was handled for both the coagulant (nopal) and flocculant (cassava starch).

#### 2.3.3 Statistical analysis

Descriptive data were calculated and a test of variance analysis (ANOVA) for comparing simple averages was applied, verifying the model assumptions; in case of default, the Kruskal Wallis test was used (Navidi, 2006). The statistical software Statgraphics Centurion (version 18) was used in this research.

## 3 Results and discussion

**Figure 1:**
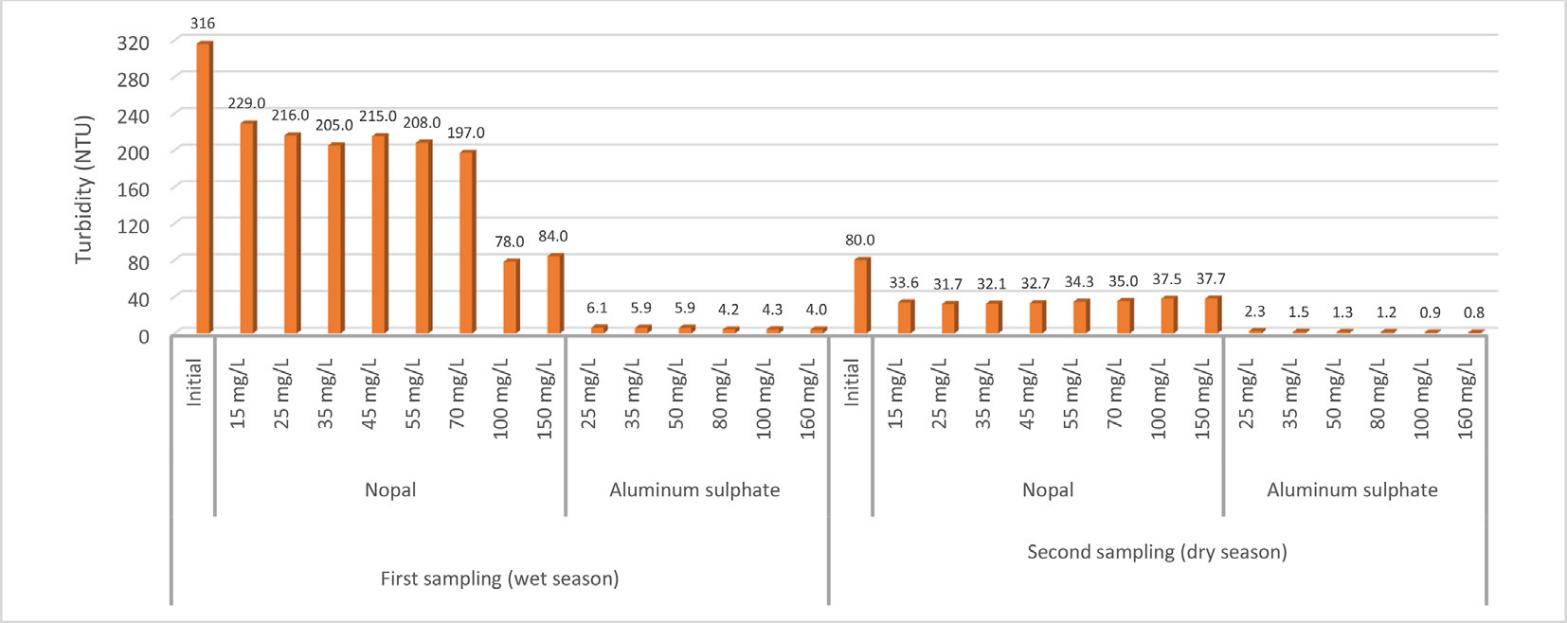
Variation of turbidity with the application of test jars in the two samplings.

**Figure 2:**
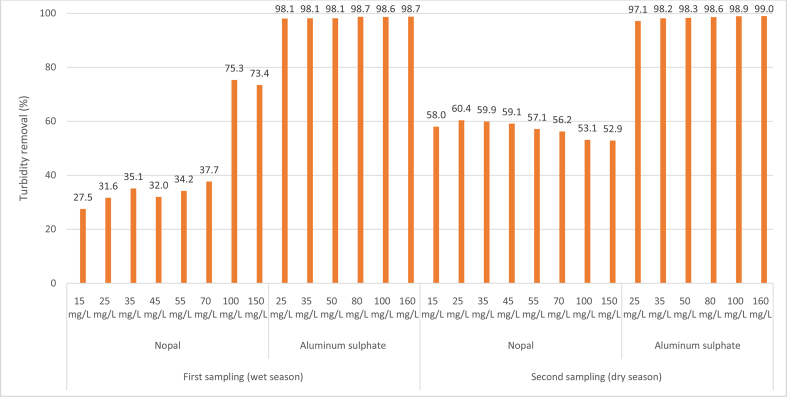
Reduction of turbidity removal with the application of test jars in the two samplings.

The optimal dose of aluminum sulfate was 160 mg/L in the two samples (Figure 1), representing an average turbidity removal effectiveness of 98.37% and a standard deviation of 0.51% (Table 1 and Figure 2), the latter indicating the homogeneity of the data. The results using aluminum sulfate turbidity test jars ranged between 4 and 6.1 NTU in the first test (wet season); while in the dry season, the results between 0.8 and 2.3 NTU, indicating that in the latter the chemical coagulant had the lowest values of this parameter. This could be related to the initial turbidity without treatment that was 80 NTU, while turbidity during the rainy season was 316 NTU (Figure 1).

**Table 1:** Descriptive statistics of the efficiency of turbidity removal (%) of the coagulants studied.

Coagulant	N	Average	Median	Standard deviation	Minimum	Maximum	Rank	Lower limit	Upper limit
Nopal–starch cassava combination	12	52.74	55.14	14.54	33.54	67.50	33.96	48.54	56.93
Nopal	16	50.22	54.65	15.14	27.50	75.30	47.80	46.43	54.00
Aluminum sulfate	12	98.37	98.45	0.51	97.10	99.00	1.90	98.22	98.51
Total	40	65.42	61.77	25.02	27.50	99.00	71.50	61.46	69.37

Note: All reported values are in percentages (%) except those corresponding to the number of data analyzed (N).

Experimentally, the optimum dose of nopal varied between samplings, with the first being 100 mg/L and the second, 25 mg/L. The final turbidities in the first test ranged between 78 and 229 NTU; while in the second it was between 31.7 and 37.7 NTU. On average, the removal efficiency of turbidity was 50.2% with a standard deviation of 15.14%. This differs from the efficiencies found by Olivero et al. (2014) and Miller et al. (2008), who achieved reductions in turbidity between 83.66% and 92%–99%, respectively, using the nopal as a natural coagulant to clarify raw water; however, the methods used to extract the nopal mucilage were different from our study, and this most likely significantly affected the final result regarding effectiveness of the coagulant. Likewise, an investigation conducted by Mukhtar et al. (2015) also achieved higher removals (i.e., up to a 91% reduction in turbidity) but other species of cactus were used, specifically *Opuntia stricta*, which would make it necessary to investigate the efficacies of other species of cactus native to Colombia, such as *Melocactus curvispinus Pfeiffer subsp. Obtusipetalus*, which has not been explored in Colombia or anywhere else in the world.

On the other hand, significant differences were found between the percentages of removal via application of nopal and aluminum sulfate with a confidence level of 95% using the comparison of medians of Kruskal Wallis (P-value = 8.18 × 10^−6^) (see Table 2) as a criterion. The median value for aluminum sulfate was 98.45% and the confidence limits were 98.37% ± 0.14%, which were higher than the median value of nopal that was 54.65% and with limits of 50.22% ± 3.80 % (Table 1). This confirms reasons for the preferred use of aluminum sulfate in the world. However, it is necessary to make a greater effort to achieve more effectiveness in removing suspended particles given that other variables affect the coagulation process (e.g., pH, fast and slow mixing time, and speed gradient among other variables).

**Table 2:** Comparison of averages or medians between periods and parameters evaluated for the coagulants studied.

Comparison	Coagulant	Test	P-value
Turbidity removal (Dry and rainy Season)	Nopal–starch cassava combination	Kruskal Wallis	0.0039
	Nopal	Kruskal Wallis	0.093
	Aluminum sulfate	ANOVA	0.92
Turbidity removal	Nopal	Kruskal Wallis	8.18 × 10^−6^
	Aluminum sulfate		
	Nopal–starch cassava combination	Kruskal Wallis	3.14 × 10^−5^
	Aluminum sulfate		
	Nopal–starch cassava combination	Kruskal Wallis	3.90 × 10^−6^
	Nopal		
	Aluminum sulfate		
	Nopal–starch cassava combination	Kruskal Wallis	0.43
	Nopal		
pH	Aluminum sulfate	Kruskal Wallis	7.88 × 10^−6^
	Nopal		
	Nopal–starch cassava combination	Kruskal Wallis	3.02 × 10^−5^
	Aluminum sulfate		
	Nopal–starch cassava combination	Kruskal Wallis	3.57 × 10^−6^
	Nopal		
	Aluminum sulfate		
	Nopal–starch cassava combination	Kruskal Wallis	0.54
	Nopal		

The reason that aluminum sulfate is more efficient than prickly pear may be because in the Al(OH)_x_ species, aluminum interacts with colloids and natural organic matter (NOM) via hydrogen bonding and electrostatic interaction; the precipitation of amorphous metal hydroxide that occurs with the addition of aluminum salts to the influent in the coagulation process strongly adsorbs organic substances, presenting charge neutralization (i.e., Al hydroxides have a positive charge and NOM have negative charges) at pH between 5 and 6, the range of neutrality in which natural water sources are usually found (Duan and Gregory, 2003). While the nopal would prefer to interact through two main mechanisms: 1) by electrostatic affinities in the case of the presence of groups of quaternary amines available in the influent; and 2) via hydrogen bonding due to the hydroxyl or carboxyl groups that naturally occur on the backbone (Lapointe and Barbeau, 2019).

On the other hand, the optimal coagulant activity of nopal occurs in basic waters (8 < pH < 10) (Miller et al., 2008), a range well above that of the study source (the Magdalena River), so pH could have influenced the efficiency of nopal as a coagulant. Additionally, possible biodegradation of prickly pear would also affect turbidity removal performance in water compared to the evaluated synthetic coagulant (i.e., aluminum sulfate).

However, aluminum sulfate encountered problems in significantly reducing the pH, ranging between 3.9 and 6.1 units, due to the aluminum salts reacting with the water alkalinity (AWWA, 1999). This is a serious problem in treatment plants due to the additional cost incurred to correct this problem with the application of alkalizing before dosing chemical compounds, in addition to the issues related to the corrosion of water treatment system components due to low pH.

**Figure 3:**
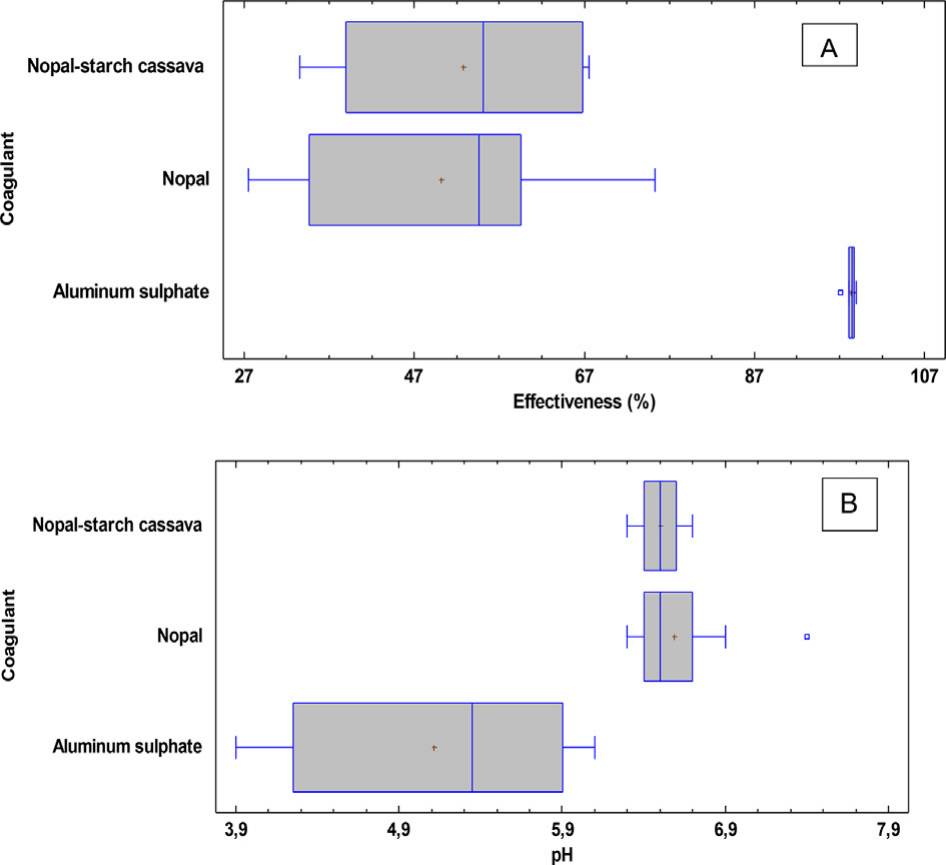
Boxplot of the parameters evaluated. A) Turbidity removal efficiency between coagulants. B) Variation of pH between coagulants.

Table 1 shows that the efficiency in turbidity removal using aluminum sulfate was the most homogeneous (see Figure 3A) with a standard deviation of 0.51%, while results of the nopal and nopal–cassava starch combination had standard deviations of 15.14% and 14.54%, respectively. This indicates that, with the doses applied with aluminum sulfate, its effectiveness was not affected during the removal of suspended solids from the water study in the two meteorological sampling seasons (i.e., wet and dry), while the variation of the parameters evaluated if aluminum sulfate could affect the performances of the natural coagulant or the coagulant–flocculant mixture (i.e., nopal and cassava starch).

Considering the above, a comparison of averages or medians was applied to determine if there was variation between the efficiency of the coagulants with respect to the meteorological time of sampling (i.e., dry and rainy seasons). The results revealed that both nopal and aluminum sulfate had the same performance during the two sampling periods concerning turbidity reduction in the studied water (P-value> 0.05) (see Table 2); while the nopal–cassava starch combination showed significant statistical variations (P-value = 0.0039). Thus, it would be appropriate to carry out a study that evaluated the incidence of climatic variability on the efficiency of the coagulant–flocculant mixture explored in the present paper.

Additionally, comparisons between the nopal and nopal–cassava starch combination with aluminum sulfate were analyzed for turbidity removal of the mass of water treated with these coagulating substances. In these tests, it was found that there were significant differences between all the comparisons with aluminum sulfate (P-value <0.05), while no differences were presented in the combination mixture of nopal–cassava starch and nopal (P-value = 0.43) (see Table 2), indicating in the latter that the performance between these two presentations of natural coagulants was similar.

On the other hand, it is highlighted that starch-based products are capable of acting simultaneously as a coagulant and flocculant as long as there is a process of carboxymethylation or phosphorylation in their chemical structures to induce an increase in anionic charge density and molecular weight, which allows potentiating the mechanisms of: 1) electrostatic interactions between colloids and anionic carboxymethyl groups; and 2) via hydrogen bonding between oxygen double bonds and silanol, aluminol, and metal hydroxides (Lapointe and Barbeau, 2019). It is important to note that one of the disadvantages of native starches (including cassava starch) is that they have low molecular weight and are poorly soluble without carboxymethylation or phosphorylation (Lapointe and Barbeau, 2019; Bolto and Gregory, 2007), which could influence its performance as a flocculant in this study, therefore, future research should focus on improving the performance of these combinations of natural flocculant coagulant for water treatment so that they are as effective as aluminum sulfate.

However, aluminum sulfate presented significant problems in pH reduction, ranging between 3.9 and 6.1 units (see Figure 3B), which is because aluminum salts react with water alkalinity (AWWA, 1999). This is a serious problem in treatment plants due to the additional investment cost for its correction, the application of alkalizing agents prior to the dosing of coagulating compounds, as well as low pH that corrodes the components of the drinking water treatment system.

**Figure 4:**
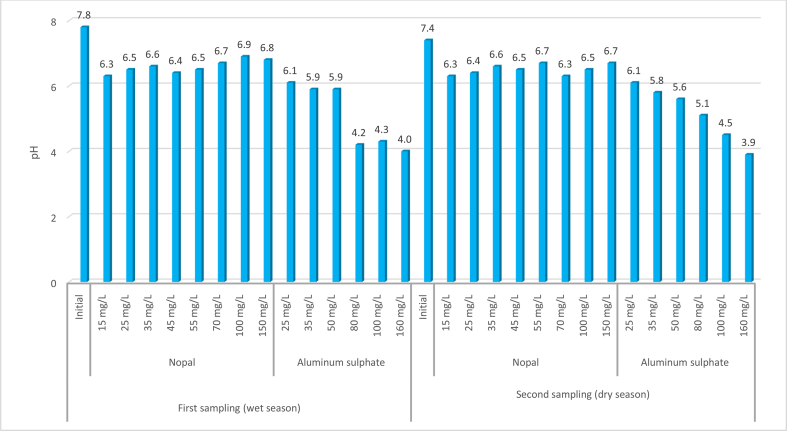
Variation of the pH with the application of test jars in the two samplings. The pH value is presented on the Y-axis. The following is indicated hierarchically in the X-axis: The two samples carried out in two sample periods (wet season and dry season), the coagulant used (nopal and aluminum sulfate), the doses in mg/L of each coagulant and, additionally, the value of the sample before treatment (Initial). Finally, the values shown in the figure refer to the pH after the coagulation–flocculation process compared to the pH of the raw water (Initial).

The pH results of the nopal mucilage coagulant show that variations were minimal and were maintained near neutrality after the application of nopal, ranging between 6.3 and 6.9 pH values (see Figure 4), which was inside the Colombian standard for quality water (Resolution 2115, 2007), which states that the pH range must be 6.5 to 9. These small pH variations coincide with those reported by Olivero et al. (2013) and Villabona et al. (2013), which is an advantage over aluminum sulfate and would not require additional operations or processes to neutralize the pH in a treatment water plant. This finding indicates potential decreased operation costs, as well as other socio-environmental benefits that these natural compounds provide (e.g., low cost, safety with regards to human health, and biodegradation potential) (Lugo and Lugo, 2018).

Moreover, the combined test results are very interesting because corroborating a flocculation assistant like cassava starch increases the effectiveness of mucilage nopal as a coagulant water clarification process without severely affecting the pH of the water to be treated, as it only varied between 6.3 and 6.7 (see Table 3). Table 2 shows that there were significant differences in the final pH, after the application of coagulants, between the presentations of natural coagulants against aluminum sulfate (P-value <0.05), while the comparison of nopal against the nopal–cassava starch combination showed no differences (P-value = 0.54).

**Table 3:** Effectiveness nopal–cassava starch combination.

Sampling	% combination	Dose mL/L	pH Initial	Initial Turbidity (NTU)	Final pH	Final Turbidity (NTU)	% Effectiveness
first sampling	Nopal 93	Nopal 6.5	7.8	316	6.3	200	36.71
	starch 7%	starch 0.5					
	Nopal 85%	Nopal 6	7.8	316	6.5	208	34.18
	starch 15%	starch 1					
	Nopal 75%	Nopal 5.2	7.8	316	6.6	180	43.04
	starch 25%	starch 1.8					
	Nopal 73%	nopal 5.1	7.8	316	6.4	186	41.14
	starch 27%	starch 1.9					
	Nopal 67%	Nopal 4.7	7.8	316	6.5	167	47.15
	starch 33%	starch 2.3					
	Nopal 60%	Nopal 4.2	7.8	316	6.7	210	33.54
	starch 40%	starch 2.8					
second sampling	Nopal 93%	Nopal 2.3	7.4	80	6.3	29,5	63.13
	starch 7%	starch 0.2					
	Nopal 85%	Nopal 2.1	7.4	80	6.5	26.37	67.04
	starch 15%	starch 0.4					
	Nopal 75%	Nopal 1.8	7.4	80	6.6	26.7	66.63
	starch 25%	starch 0.7					
	Nopal 73%	Nopal 1.7	7.4	80	6.4	26.5	66.88
	starch 27%	starch 0.8					
	Nopal 67%	Nopal 1.6	7.4	80	6.5	26	67.5
	starch 33%	starch 0.9					
	Nopal 60%	Nopal 1.5	7.4	80	6.7	27.3	65.88
	starch 40%	starch 1					

Note: starch refers to cassava starch.

In Table 3, a similarity can be observed in the two tests when the combination of 67% nopal and 33% cassava starch were used, which results in the two samples with a higher percentage of effective turbidity removal compared to the remaining combinations, these being 47.15% in the first test and 67.5% in the second. Thus, this combination is considered optimal for laboratory tests on the source of study water and should be considered for further evaluation in the field.

As a final laboratory test, the dry sampling, nopal–cassava starch combination was evaluated in the TA- CF-005/PE equipment, and we found that turbidity values ranged from 47 to 75 NTU after the coagulation–flocculation–sedimentation process, representing 41.25% and removals at 6.25%, respectively (see Table 4); while the pH was maintained between 6.4 and 6.8 (see Table 4), confirming that the mixture of these two compounds does not affect the natural pH, as determined in the test jar. The efficiency was lower in the compared equipment jars, which may be because the flows administered by dosing coagulant and flocculant pumps were disproportionate to their respective dosages, since the flow product and coagulant concentration represents the load of substance applied to the body of water to be treated (tributary) and affects the performance of the continuous water flow treatment plants.

**Table 4:** Variation of equipment efficiency TA- FQ-005/PE.

Initial Turbidity (NTU)	pH Initial	Measurement Time (minutes)	Raw Water Flow Rate (L/h)	Flow Coagulant Dosage (L/h)	Flocculent Dosing Flow (L/h)	Final Turbidity (NTU)	Turbidity Removal (%)	Final pH
80	7.4	5	10	0.7	0.5	54	32.5	6.5
80	7.4	10	5	0.4	0.1	47	41.3	6.8
80	7.4	15	8	0.3	1	61	23.8	6.4
80	7.4	20	12	3	2	75	6.3	6.6

The turbidity value in NTU is presented on the Y-axis. The following is hierarchically indicated in the X-axis: The two samples carried out in two sample periods (wet season and dry season), the coagulant used (nopal and aluminum sulfate), the doses in mg/L of each coagulant and additionally, the value of the water sample before treatment (Initial). Finally, the values shown in the figure refer to the turbidity measured after the coagulation–flocculation process compared to the turbidity of the raw water (Initial).

The value of the efficiency in the removal of turbidity in percentage (%) is presented on the Y-axis. The following is hierarchically indicated in the X-axis: The two samples carried out in two sample periods (wet season and dry season), the coagulant used (prickly pear and aluminum sulfate), and the doses in mg/L of each coagulant. Finally, the values shown in the figure refer to the percentages (%) of turbidity reduction after the coagulation–flocculation process.

## 4 Conclusions

Nopal, in combination with cassava starch at concentrations of 1%, 67%, and 33%, respectively, increases their combined effectiveness at an average of 8.5%. The concentrations used only for coagulant (nopal) and combined with the flocculant (cassava starch) showed high removals, 60.4% and 67.04%, respectively. For future studies, we recommended that efforts should be made to improve the chemical characteristics of these natural substances to strengthen the coagulation–flocculation mechanisms in order to increase the effectiveness of this natural mixture evaluated in this article.

However, it is important to consider the approach of using two or more natural compounds that would solve the issue of using a single biomass (monoculture), which can cause severe environmental impacts. Additionally, the amounts that can be obtained from crops are not always representative of projecting these investigations to a decentralized treatment plant or to a municipality.

Furthermore, the mucilage nopal alone, as well as mixed with cassava starch, did not significantly affect the pH and maintained values within the expected norm. This is an advantage over aluminum sulfate that acidified water. It is a disadvantage with regard to natural water because it requires neutralizing solutions, which will incur additional costs in the water purification process.

Finally, the equipment for the physical–chemical treatment study at a small-scale with reference (Prominent TA-CF-005/PE) can recreate the physical–chemical conditions of a water treatment plant in real time and measure water quality variables at the same time varying operation conditions and thus, accurately study the effect of these on the water to be treated. Therefore, this procedure should be considered to be an important tool in the development of academic and research practices for University of Cundinamarca as well as the Colombian and global context as an alternative to test jars.

## Declarations

### Author contribution statement

José Lugo-Arias: Conceived and designed the experiments; Analyzed and interpreted the data; Wrote the paper.

Elkyn Lugo-Arias: Analyzed and interpreted the data; Wrote the paper.

David Ovallos-Gazabon: Conceived and designed the experiments; Performed the experiments; Contributed reagents, materials, analysis tools or data.

Juan Arango: Conceived and designed the experiments; Performed the experiments; Analyzed and interpreted the data.

Mario de la Puente: Contributed reagents, materials, analysis tools or data; Wrote the paper.

Jesús Silva: Conceived and designed the experiments; Analyzed and interpreted the data; Contributed reagents, materials, analysis tools or data.

### Funding statement

This research did not receive any specific grant from funding agencies in the public, commercial, or not-for-profit sectors.

### Competing interest statement

The authors declare no conflict of interest.

### Additional information

No additional information is available for this paper.
